# The Development of Metal-Free Porous Organic Polymers for Sustainable Carbon Dioxide Photoreduction

**DOI:** 10.3390/nano14171432

**Published:** 2024-09-02

**Authors:** Ranjit Bariki, Reshma G. Joseph, Oussama M. El-Kadri, Mohammad H. Al-Sayah

**Affiliations:** 1 Materials Science and Engineering Program, College of Arts and Sciences, American University of Sharjah, Sharjah P.O. Box 26666, United Arab Emirates; 2 Department of Biology, Chemistry and Environmental Sciences, American University of Sharjah, Sharjah P.O. Box 26666, United Arab Emirates; 3 Materials Research Centre, College of Arts and Sciences, American University of Sharjah, Sharjah P.O. Box 26666, United Arab Emirates

**Keywords:** POPs, photoreduction, carbon dioxide, metal-free, porous material, organic polymers, microporosity

## Abstract

A viable tactic to effectively address the climate crisis is the production of renewable fuels via photocatalytic reactions using solar energy and available resources like carbon dioxide (CO_2_) and water. Organic polymer material-based photocatalytic materials are thought to be one way to convert solar energy into valuable chemicals and other solar fuels. The use of porous organic polymers (POPs) for CO_2_ fixation and capture and sequestration to produce beneficial compounds to reduce global warming is still receiving a lot of interest. Visible light-responsive organic photopolymers that are functionally designed and include a large number of heteroatoms and an extended π-conjugation allow for the generation of photogenerated charge carriers, improved absorption of visible light, increased charge separation, and decreased charge recombination during photocatalysis. Due to their rigid structure, high surface area, flexible pore size, permanent porosity, and adaptability of the backbone for the intended purpose, POPs have drawn more and more attention. These qualities have been shown to be highly advantageous for numerous sustainable applications. POPs may be broadly categorized as crystalline or amorphous according to how much long-range order they possess. In terms of performance, conducting POPs outperform inorganic semiconductors and typical organic dyes. They are light-harvesting materials with remarkable optical characteristics, photostability, cheap cost, and low cytotoxicity. Through cocatalyst loading and morphological tweaking, this review presents optimization options for POPs preparation techniques. We provide an analysis of the ways in which the preparative techniques will affect the materials’ physicochemical characteristics and, consequently, their catalytic activity. An inventory of experimental methods is provided for characterizing POPs’ optical, morphological, electrochemical, and catalytic characteristics. The focus of this review is to thoroughly investigate the photochemistry of these polymeric organic photocatalysts with an emphasis on understanding the processes of internal charge generation and transport within POPs. The review covers several types of amorphous POP materials, including those based on conjugated microporous polymers (CMPs), inherent microporosity polymers, hyper-crosslinked polymers, and porous aromatic frameworks. Additionally, common synthetic approaches for these materials are briefly discussed.

## 1. Introduction

**Figure 1:**
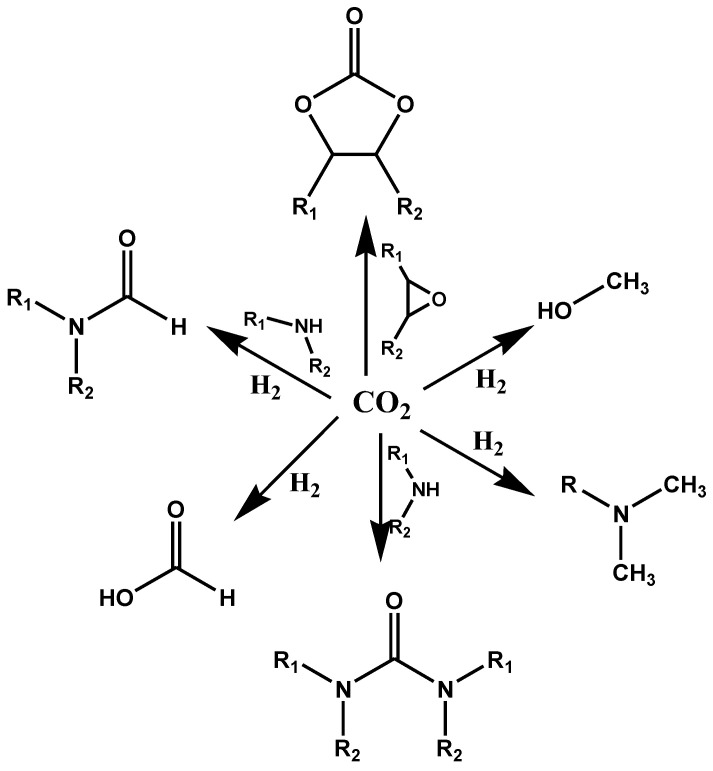
Graphical illustration of CO_2_ conversion pathways.

It is projected that global energy consumption will increase by 28% by 2040. Currently, over 80% of the global energy demands are met by the burning of non-renewable fossil fuels, like coal, petroleum, and natural gas, which is responsible for CO_2_ concentration in the atmosphere [1,2]. Following the Industrial Revolution, atmospheric CO_2_ levels have increased from 280 ppm to 400 ppm, with projections suggesting it could reach 500 ppm by the 22nd century. Such an elevation is anticipated to elevate the Earth’s mean temperature by 1.9 °C. The continuously increasing anthropogenic CO_2_ emissions are considered the primary cause of the greenhouse gas effect, leading to phenomena such as global warming, ice melting, and rising sea levels, among other environmental challenges [3]. If not effectively addressed, global warming could escalate rapidly in the future. However, with proper intervention and management, Earth could eventually transition into a hospitable planet for human life, mitigating the risks associated with climate change [4,5]. Among them, the CO_2_ capture method, as a major C1 source, not only mitigates the greenhouse effects but also acts as an effective synthetic method for several chemicals. CO_2_ can be converted to useful products using various pathways, as represented in Figure 1 [6].

The CO_2_ molecule exhibits both chemical and thermal stability, making it resistant to conversion into other chemicals under mild conditions. Alternatively, CO_2_ activation can be facilitated by using an effective catalyst [6]. It can be chemically transformed by thermo-, electro-, or photocatalytic conversion. Table 1 presents some of the catalysts used for CO_2_ reduction. The electrocatalytic reduction of CO_2_ can yield diverse products, including CO, formate, formaldehyde, methane, ethylene, alcohols, and more. However, attaining high selectivity toward a single product remains challenging, particularly concerning hydrocarbons with two carbon atoms or more (C2+ hydrocarbons) and oxygenated hydrocarbons. In addition, CO_2_ can be transformed into eco-friendly solar fuels, like CH_4_, HCO_2_H, CH_2_O, and CH_3_OH, through photocatalytic reduction [7]. This chemical conversion can also be accomplished through cycloaddition reactions of epoxides and propargylic alcohols with CO_2_, the reductive conversion of CO_2_ with H_2_, and photocatalytic/electrocatalytic processes [8].

Molecular photocatalysts, encompassing inorganic materials, metal complexes, and organic dyes, have been extensively studied in the field of photocatalysis. However, the effectiveness of these catalysts is hampered by various drawbacks, such as metal toxicity, poor stability, and difficulty in separation. Diverse substrates have been explored as heterogeneous supports for molecular photocatalysts to overcome these limitations and enhance catalytic performance and practical utility. These substrates include glass, fabrics, and polymers. Organic polymers stand out because they are stable, non-toxic, cost-effective, and processable. Nonetheless, challenges persist, such as low dispersity of solvents, limited interaction between catalysts and substrates, and restricted exposure of active sites in conventional polymers, thereby constraining overall efficiency and activity [27].

## 2. Fundamentals of Photocatalytic CO_2_ Reduction

**Figure 2:**
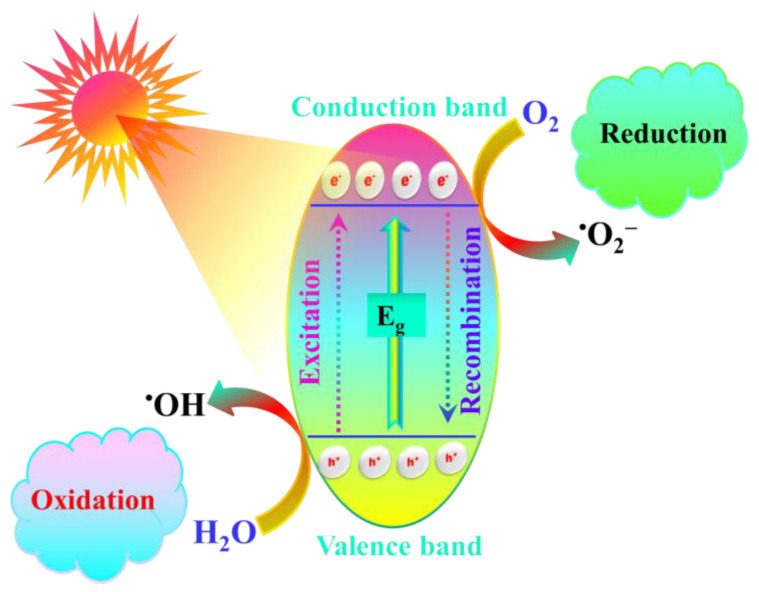
Working principle of a semiconductor photocatalyst [29].

The working principle of a semiconductor photocatalyst (schematically shown in Figure 2) is based on the excitation of valence electrons toward the conduction band when the light of definite energy falls on the photocatalytic surface. This movement is possible only if the energy of the incident light matches or is greater than the band energy gap between the valence band and the conduction band. As a result, holes are generated in the valence band, which can oxidize donor molecules, such as water molecules, to produce hydroxyl radicals. These radicals exhibit potent oxidizing properties, pivotal in pollutant degradation [27,28]. Meanwhile, the electrons in the conduction band lead to the reduction of acceptors, such as oxygen molecules, forming highly reactive radical anions.

CO_2_ is thermodynamically stable and unreactive in the gas phase. The energy gap between the highest occupied molecular orbital (HOMO) and the lowest unoccupied molecular orbital (LUMO) of CO_2_ is 13.7 eV, with an electron affinity of approximately 0.6 ± 0.2 eV [29,30]. Consequently, the redox potential for one-electron transfer in the reduction reaction of CO_2_ (Equation (1)) is highly negative (E° redox = 1.90 eV vs. NHE) [3,19]. The considerable energy required for electron transfer to produce active species for CO_2_ conversion presents a significant challenge, making both photocatalytic and electrocatalytic processes exceptionally demanding tasks [31]. It needs high temperature, pressure, and suitable catalysts to become activated and react. Hence, the presence of basic sites on the catalyst surfaces helps to adsorb and activate the CO_2_ molecules, since CO_2_ is acidic [6]. As the absorption of light by the photocatalyst generates holes in the valence band and electrons in the conduction band, the activated CO_2_ molecules are reduced by the electrons generated in the conduction to the radical anions (Chemical Equation (1)) [2,32].



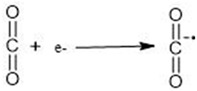

(1)


**Figure 3:**
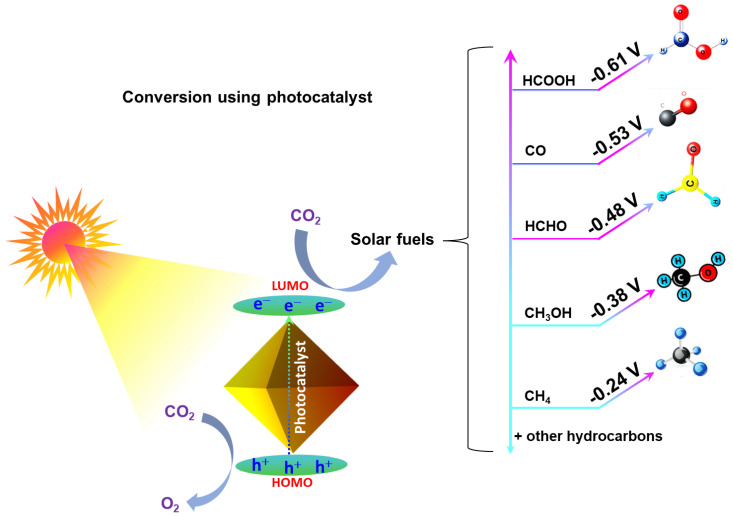
Graphical illustration of photocatalytic CO_2_ reduction to solar fuels.

These radical anions of CO_2_ can undergo further reactions to produce hydrocarbons (Figure 3), but a reducing agent that supplies hydrogen (such as H_2_O, H_2_, CH_4_, and CH_3_OH) is required [1,33]. The non-carbonic sources, such as H_2_O and H_2_, form C1 products with added economic value, such as carbonate, carbamate, or urea [34]. It can also be converted into C2 products, such as C_2_H_6_, C_2_H_4_, and CH_3_CH_2_OH, through C-C coupling reactions [35,36].

The efficiency and product selectivity of the photocatalytic CO_2_ reduction processes are influenced by several parameters of the process [8,37]:

1. Light excitation characteristics: This encompasses properties of incident light, including wavelength, intensity, and polarization. These factors influence the generation of photogenerated electrons;
2. Band structure: The electronic band structure of the catalyst material dictates the energetics of electron-transfer processes, impacting the reactions that can occur;
3. Effectiveness of separating photogenerated charge carriers: Efficient separation of photogenerated electrons and holes is essential to prevent recombination, maximizing the utilization of photogenerated carriers for catalysis.

**Figure 4:**
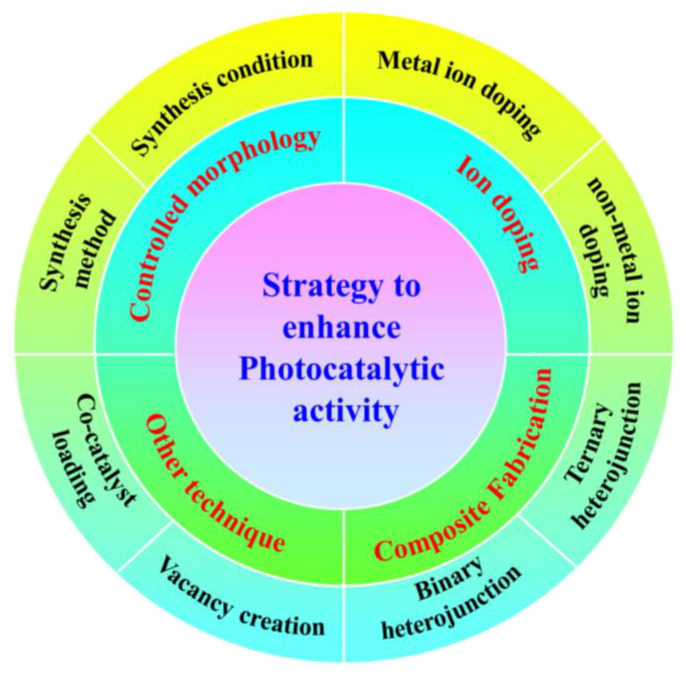
Schematic model of various strategies to improve photocatalytic efficiency of metal-free POPs.

The light-absorption capacity of a photocatalyst is determined by the band-gap energy, representing the potential variance between the valence and conduction bands. Materials with narrower band gaps, such as POPs, exhibit heightened light-absorption efficiency. POPs have narrow band gaps due to their highly conjugated structures, and this band gap is tunable through structural variations (Figure 4) [38]. In addition, incorporating donor–acceptor structures in the structure of POPs helps to overcome the limitations associated with charge-carrier recombination, thereby boosting the efficiency and effectiveness of the photocatalytic process. Finally, the presence of heteroatoms with the structure of POPS and their porous structures provides basic sites to adsorb and activate targeted molecules, such as CO_2_ molecules [6,35]. Therefore, POPs have great potential to be effective photocatalysts for CO_2_ reduction.

## 3. Structure and Properties of POPs

Porous materials can be categorized based on the pores’ size, the building blocks’ structure, and the level of long-range order. The IUPAC convention divides porous materials into three categories, namely (i) microporous, which has a pore diameter of less than two nanometres; (ii) mesoporous, which has a pore diameter of between two and fifty nanometres; and (iii) macroporous, which has a pore diameter greater than fifty nanometres [39,40,41]. Porous materials can be classified into three subclasses based on their building blocks, namely (i) inorganic, (ii) inorganic–organic hybrid (e.g., metal–organic frameworks), and (iii) organic porous materials (e.g., porous organic polymers, porous molecular solids, and H-bonded organic frameworks, HOFs). [39,41]. Some of these organic porous materials are amorphous, while others have crystalline frameworks, commonly referred to as covalent organic frameworks (COFs) [42]. COFs are covalently bonded organic porous polymers with periodic organization, permanent porosity, and chemical and thermal durability. Due to their ease of characterization, COFs are widely used in sensing, gas absorption/storage, and catalysis [43,44,45].

**Figure 5:**
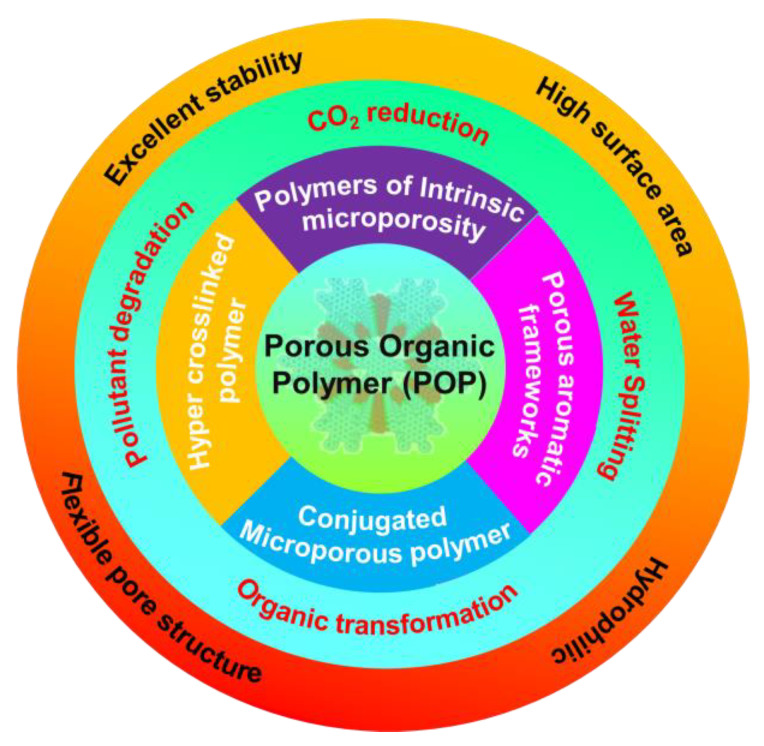
Classification, properties, and photocatalytic application of POPs.

Researchers have focused a lot of interest on POPs, a developing organic porous material, because of their high porosity, exceptional chemical stability, structural adjustability, and range of features [46]. These days, amorphous POPs are the subject of numerous reviews, but only a small number of them methodically outline how they are used in photocatalytic applications (Figure 5). To comprehend the current state of heterogeneous photocatalysts in photocatalytic organic transformations, various reported amorphous POPs are systematically categorized in this research. The paper will begin with a detailed overview of the building blocks of a number of popular amorphous POPs. Following that, the common types of photosynthesis will be categorized based on the reactions identified by earlier reviews and investigations. Finally, a summary of the current state of development in the field of photocatalytic application based on amorphous POPs will be provided, along with potential issues that may arise and opportunities for further study.

**Figure 6:**
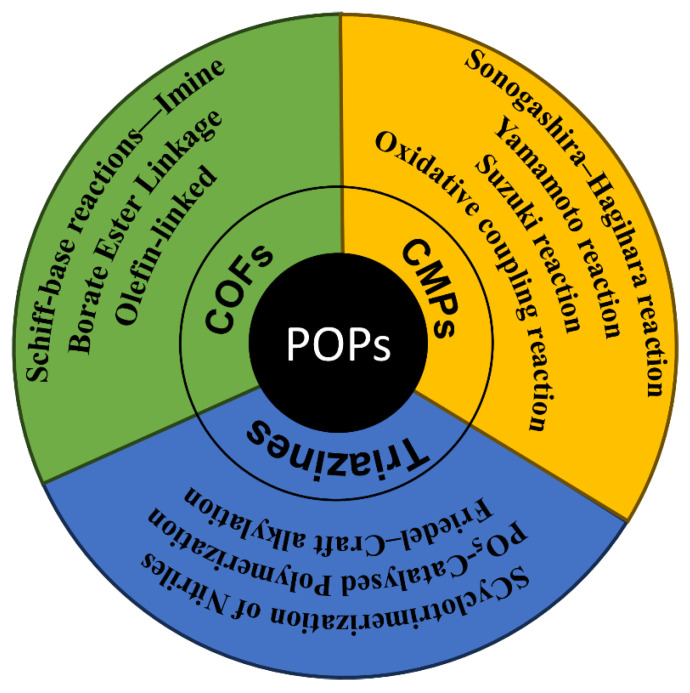
Summary of synthetic approaches for preparation of POPs.

Heterogeneity is the most alluring feature of POPs as catalysts, since it gives them significant advantages in separation and cyclic reaction utilization. High stability is necessary for POPs to retain their porosity structure and catalytic activity after numerous repurposings. POPs can endure the catalytic environment because of the covalent bond, a durable chemical connection. Many strategies for developing such strong structures have been reported and reviewed before [47,48,49]. Figure 6 provides a schematic summary of the different synthetic approaches for preparing POPs. In general, the synthetic approach is based on the composition of the building blocks and intended applications. CMPs with a microporous network of a p-conjugated system and high chemical stability are often produced through C–C coupling reactions like the Sonogashira–Hagihara reaction, Yamamoto reaction, Suzuki reaction, and oxidative coupling reaction [27,50,51]. The strongly-bonded covalent triazine frameworks (CTFs), however, are prepared through other reactions, such as phosphorus pentoxide catalyzed polymerization, ionothermal cyclotrimerization of nitriles, and Friedel–Craft alkylation, among others [52,53,54,55,56,57].

On the other hand, some COFs are created using reversible bonding. Due to their regular pores and organized framework, COFs have potential applications across various industries. Generally, COFs exhibit good chemical stability, but their stability is greatly impacted by their functional groups. For example, the imine-attached COFs, produced via Schiff-base reactions between amines and aldehydes, are significantly more stable than COFs with borate ester connections between a boronic acid and a catechol [27,58,59]. While imine-based COFs are not stable under strong acid conditions, their stability in harsh conditions can be significantly enhanced by converting imine moieties into thiazole. Finally, another method to produce stable COFs is to directly fabricate olefin-linked COFs that have full α-conjugated frameworks and exhibit semiconductive abilities [60,61].

POPs also demonstrate exceptional photo and thermal stability. Conjugated polymers, including CMPs and CTFs, contain tightly bonded chemical structures that enhance their thermodynamic stability. The mass fall of CMP-1, which is made of 1,3,5-triethynylbenzene and 1,4-diiodobenzene, is not evident until the temperature rises over 400 °C in an environment of nitrogen [59,62]. Under oxygen, the 5-(4-cyanophenyl)thiophene-2-carbonitrile-prepared CTF shows excellent thermal stability up to 500 °C. COFs typically decompose at temperatures below 350 °C. Benzoxazole-linked COFs have exceptional photostability and are thermally stable to 400 °C. In addition, the crystalline frameworks retain their integrity after at least three days of constant exposure to visible light. POPs’ ultra stability ensures that they can be used again in photocatalytic reactions without causing toxicity or secondary pollution. Additionally, POPs have great potential for catalyzing high-temperature processes, such as partial methane oxidation and ethylene oxidation, due to their heat stability.

POPs exhibit interesting features that make them a reliable and efficient catalyst widely used in various applications. Their porous framework has a high surface area, making them ideal for adsorption, catalysis, and gas storage [27]. They are primarily mesoporous, can selectively adsorb molecules, and are useful for water purification, environmental cleanup, and separation processes. POPs excel in carbon capture, hydrogen storage, and gas purification due to their efficient gas storage and separation capabilities [63]. They demonstrate excellent hydrothermal stability and structural versatility and are suitable for drug delivery, sensing, and membrane separation. Although amorphous and lightweight, POPs offer excellent composition control and high specific surface areas, enhancing their performance in catalytic transformations. They stabilize metal nanoparticles, preventing aggregation and leaching during catalytic operations [64].

## 4. POPs in Photocatalysis

Intrinsically semiconductive POPs are activated when exposed to visible light with an energy equal to or greater than the bandgap. This activation leads to the production of photoexcited charge carriers, which are photogenerated electrons and holes in the valence band (VB) and conduction band (CB), respectively [6,65]. The reaction between POPs and substrates is activated by efficient charge separation and the right band potential alignment. If most of the electrons and holes created by photogeneration recombine, the efficiency of photocatalysis could temporarily decrease. Various techniques have been studied to prevent recombination and enhance charge separation in POPs. These techniques include using electron donor–acceptor systems in the structural design, asymmetrical structures, high crystallinity and coplanarity polymers, hybridization, and the introduction of noble metals [1,66]. Incorporating metals into polymers with inherent binding sites is not complicated. For instance, a pyridine-based CTF, including attached rhenium, can catalyze the photoreduction of CO_2_, exhibiting enhanced catalytic activity and improved electron–hole separation. Several factors influence the efficiency of photocatalysis in polymeric organic photovoltaics. The separation of photogenerated electrons and holes can be enhanced by using a donor–acceptor (D-A) system in POPs made of both electron-donating and electron-accepting monomers [65,67]. The polymers’ shape and surface area impact the substrate transmission rate. Variations in substituents and chemical composition can alter the energy band structure of the polymers, thereby affecting their redox potential. In photocatalytic processes, the geometry and chemical structure of the polymers are also critical factors.

### 4.1. Molecular Architectures of POPs for Photocatalysis

#### 4.1.1. Carbazole-Based Amorphous POPs

Carbazoles and their derivatives are a commonly occurring type of nitrogenous heterocyclic compounds known for their stability, π-conjugated structure, and versatile design characteristics [41,68]. Recently, carbazole and its derivatives have been used as the basic building blocks in the design and synthesis of a number of functionalized amorphous POP materials, which are widely used in photocatalysis. Wang et al. developed a novel, highly efficient POP-containing carbazole as an electron-rich center and thiophene as an auxiliary group (CTT-POP) by using a catalyst-free Schiff-base polymerization reaction [68].

**Figure 7:**
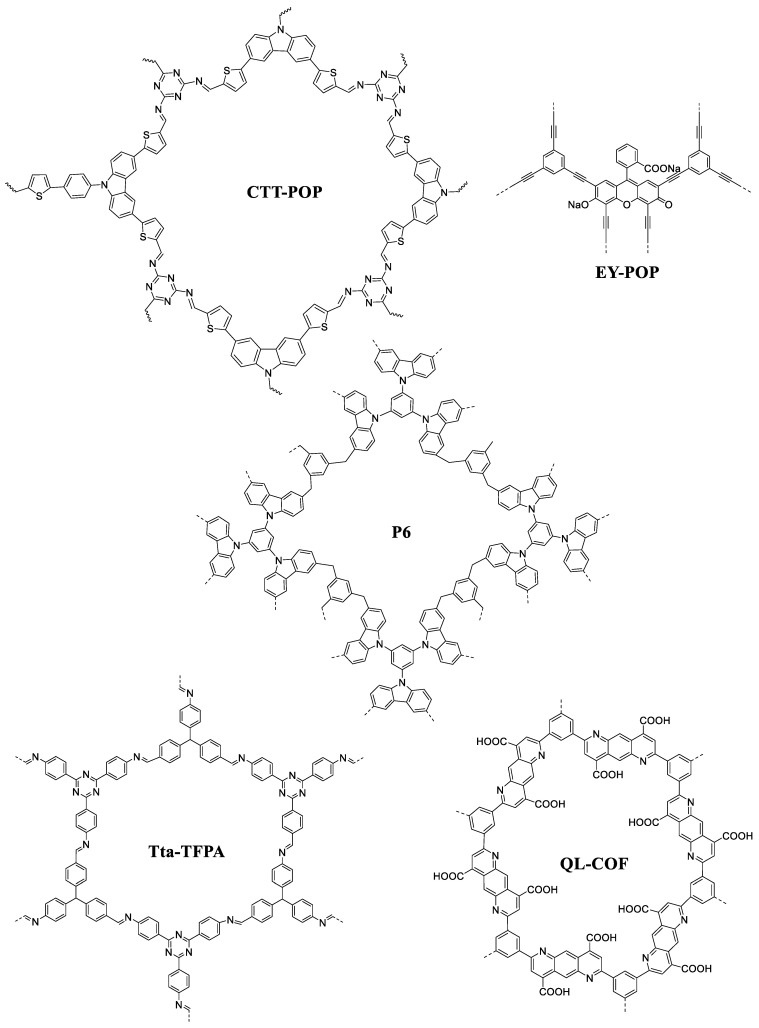
Structure of some recently reported POPs for photocatalytic reduction of CO_2_.

Later, Das et al. reported a facile template-free strategy to fabricate carbazole-derived POP (P6) composite material by the coordination of 1,3,5-tri(9-carbazolyl)-benzene (TCB) and 1,3,5-tris(bromomethyl)-benzene (TBB) as a linker (Figure 7). Here, the In_2.77_S_4_ and POP phases remain intact with each other due to induced-polarity-driven electrostatic interaction [26]. The prepared composite material exhibited excellent visible light-absorption characteristics compared to pure POP. Introducing a semiconductor into the POP moiety enhanced the photocatalytic CO_2_ reduction efficiency due to the improved space charge separation and strong resistance to the photoexcited charge recombination.

#### 4.1.2. Porphyrin-Based Amorphous POPs

Porphyrins are heterocyclic macromolecules connected by four pyrrole rings. They possess a wide *π*-conjugation system, high stability, and the capacity to absorb visible light. These properties make them useful building blocks for organic catalysts [69,70]. Their unique square coordination makes it simple to wrap metals around them to generate metalloporphyrin complexes, significantly enhancing photocatalyst performance. Zhong et al. reported porphyrin-based POP (TEPP-F-TBT) that was successfully prepared using Sonogashira coupling and studied its photocatalytic activity towards the mineralization of bisphenol A (BPA) [71]. Li et al. reported that a Py-POP, a porphyrin-based porous organic polymer, is an effective multifunctional platform that combines photocatalysis and adsorption [72]. Py-POP was synthesized using an easy bottom-up method to produce agglomerated sphere-shaped particles with diameters of around 200 nm, making it easy to disseminate in water. Due to the size/shape exclusion effect, Py-POP shows an excellent feature towards adsorption and photocatalytic degradation of methylene blue and RhB dyes.

#### 4.1.3. Pyrene-Based Amorphous POPs

Pyrene is an organic compound with a high degree of planarity and an ultralong *π*-conjugation system. It is surrounded by a large number of active sites that can replace different functional groups to generate multifunctional materials. Consequently, pyrene and its derivatives-based organic polymers have become highly effective photosensitizers for photocatalysis, particularly photocatalytic hydrogen precipitation [41,67,73]. Xu et al. developed a series of CMPs, where the donor, acceptor, and π crosslinker units are pyrene, benzothiadiazole, and benzene (biphenyl), respectively [37]. The series of D-π-A CMPs photocatalytic materials was synthesized using the Suzuki–Miyaura cross-coupling reaction catalyzed by Pd(0). Various CMPs with different polymeric structures and components were created by varying the ratio of pyrene to benzothiadiazole units and showed excellent photocatalytic activity toward the H_2_ evolution reaction. Another example of pyrene-4,5,9,10-tetraone (PT)-linked conjugated microporous polymers (PT-CMPs) was reported by Mohamed and his coworkers [74]. This PT-CMP was prepared by the Sonogashira polycondensation reaction of ethynyl pyrene (Py-T)/tetraphenylethene (TPE-T) with two pyrene-4,5,9,10-tetraone. The prepared polymers exhibited large surface-exposed active sites with well-organized pore architectures with high activity for energy storage applications.

#### 4.1.4. Organic Dyes-Based Amorphous POPs

Organic dyes have a strong redox capacity in the excited state, which makes them appealing metal-free photocatalysts of visible light. Homogeneous organic dyes do, however, have certain disadvantages, including low light utilization, laborious production, poor reproducibility, and, above all, the difficulty of changing the molecular structure of organic dyes to obtain a tuned bandgap that is appropriate for the reaction [27,41,61]. Thus, a promising approach for developing organic photocatalysts is the creation of organic dye-based POP materials by incorporating organic dyes into microporous organic polymers. Wang et al. developed a bottom-up route for preparing two eosin Y dye-based POP frameworks (EY-POPs) (Figure 7) by using the Sonogashira cross-coupling reaction. They effectively used it as a photocatalyst for an organic transformation reaction [61]. The palladium-catalyzed eosin Y dye (EY) cross-coupling reaction with various structural building blocks resulted in an easy way to produce the polymer networks.

### 4.2. POPs in CO_2_ Photoreduction

POPs for CO_2_ photoreduction can be broadly categorized into three groups, namely (i) metal-loaded POPs, (ii) metal-free POPs, and (iii) POPs requiring external photosensitizers [36]. The metal-loaded POPs pose concerns due to their potential toxicity, metal leakage, poor selectivity, and costliness, making it a pressing issue today [8]. They have limited visible light harnessing power, and most of them use organic solvents like acetone to achieve efficient photocatalytic reduction [36]. Additionally, their susceptibility to self-oxidation, gas poisoning, and photo corrosion instability has prompted researchers to explore metal-free alternatives for photocatalytic reduction [65]. Metal-free POPs can reduce CO_2_ to a single carbonaceous product in water. Furthermore, it possesses good adsorption ability and can be tailored to have specific structures [75].

Compared to disordered POPs, the ordered structure of COFs effectively reduces electron–hole recombination and charge quenching at defect sites. The extensive spread of electrons helps to capture more light effectively. It can convert CO_2_ to methanol under visible light without a sacrificial agent. By adjusting the electronic structure of COF, it is possible to modify its photophysical properties [38].

## 5. Metal-Free POPs for CO_2_ Photoreduction

### 5.1. Photoreduction Mechanisms Specific to Metal-Free POPs

Intrinsically semiconductive POPs are excited to produce photoexcited charge carriers in the HOMO and LUMO, respectively, when exposed to visible light with an energy equal to or more than the energy gap [28,76]. These charged particles can trigger chemical reactions when interacting with other substances, but most of these electrons and holes end up recombining, which lowers the efficiency of the process [27,77]. Several approaches have been studied for POPs to prevent the recombination of charges, including high crystallinity and coplanarity polymers, asymmetrical architectures, structural design with electron donor–acceptor systems, and the introduction of noble metal and heterojunction fabrication [27]. For POPs laden with precious metals, however, leaching and photobleaching are unavoidable during catalytic reactions. However, metal-free POPs have outstanding photostability and continue to be highly active over extended periods of time. The absence of metal makes the process more cost-effective and eliminates the risk of metal contamination. However, designing and optimizing structures and reaction conditions becomes more challenging. For instance, in the case of porous conjugated polymer (PCP) materials, the main building blocks are hybridized sp^2^ and *p*_z_ orbitals, which are oriented perpendicular to the polymer structure [40,78]. This causes p-delocalization and results in a reduced HOMO–LUMO band-gap energy level (Eg) [76]. PCPs’ molecular energy level can be experimentally ascertained using ultraviolet photoelectron spectroscopy in vacuum or cyclic voltammetry. The energy level of photoactive semiconductor materials (PCPs) can also be predicted using density functional theory (DFT) computational methods.

**Figure 8:**
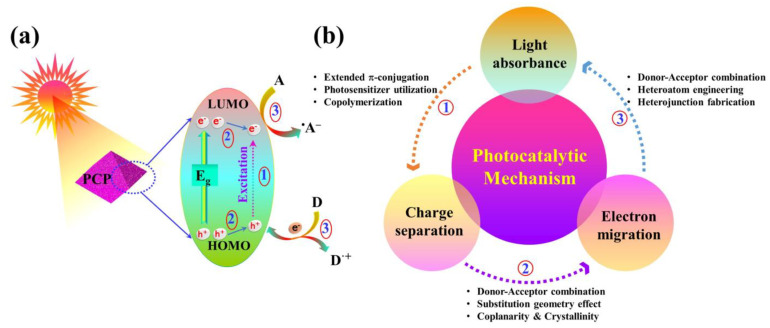
(**a**) Photocatalytic reaction pathways and (**b**) Molecular design strategy using PCP as model photocatalysts.

The process involves the creation of photo-excited electrons/holes within the material’s structure and their interaction with light, representing the initial stages of heterogeneous photocatalysis by PCPs [79]. When exposed to light, the semiconductive PCP creates electron–hole pairs by absorbing photons with higher or equal energy than its band-gap energy. Either channelization to the surface for electron/energy mobilization to substrates or (volume/surface) recombination could be the fate of the photogenerated electron and hole [40]. On the surface of PCPs, an oxidation process combines the hole with an electron from an electron source, while a reduction process donates the migrating electron to an electron acceptor. Thus, the following three phases can be regarded as critical parameters of PCPs for effective photocatalysis: (i) visible light absorption, (ii) effective dissociation of photo-excited electron–hole pairs and their channelization in PCPs, and (iii) electron or energy migration from the PCP to the substrates linked between the PCP’s band positions and the substrates’ redox potentials [62,80]. The molecular design strategy is divided into three sections according to the photocatalytic reaction mechanisms described in Figure 8.

### 5.2. Structural Features to Enhance the Photocatalytic Efficiency of Metal-Free POPs

In the design process, it is crucial to consider the internal morphology of POPs, as it greatly influences photocatalysis. With the ability to control the shape of nanoparticles, it is now feasible to deliberately create particles with specific light-related properties suitable for producing solar fuel [46,72]. The morphology can be adjusted to enhance the properties that are essential for solar fuel production, including efficient light absorption, separation of charges, optimal surface area, surface functionality, side-chain customization, and permeability to reactants such as protons. Particle structures that promote light scattering or reflection-induced re-absorption are advantageous for photocatalytic activity [69]. For example, Liu et al.‘s hollow polymer vesicles increased charge separation in thin polymer membranes and enhanced light scattering within the nanostructure by expanding the contact area, resulting in a more favorable photocatalytic activity compared to similar solid particles [67]. Compared to acceptor core–donor shell structures, an intermixed donor–acceptor blend morphology can be formed with a stronger surfactant–small molecule acceptor interaction, resulting in a more efficient charge extraction process [8]. The greater surface area in nanometer-sized (0–100 nm) particles enables more effective charge separation at the interface, removing the limitations imposed by a short exciton diffusion length in organic polymers. When PFBT nanoparticles (30–50 nm) were used in place of PFBT polymer suspension, there were five orders of magnitude increase in hydrogen evolution for PFBT polymer [46,72].

The capture and conversion of CO_2_ gas are crucial for mitigating its effect on global warming. POPs are suitable candidates for selective CO_2_ adsorption due to their limited pore-size distribution, and their electron-rich aromatic framework and prolonged conjugation provide a large surface area for strong interactions with CO_2_ molecules [46]. This local environment surrounding the polymer’s active core significantly impacts its photocatalytic activity [25,71]. Other important structural features for efficient POPs photoconversion of CO_2_ molecules are the presence of electronegative heteroatoms, like nitrogen, oxygen, or fluorine, and water wettability. The polar interactions between CO_2_ molecules and the heteroatoms help increase the CO_2_-philicity of the POPs, while the presence of water-soluble side chains enhances the process of proton reduction during the conversion of CO_2_ to hydrocarbons [81]. Finally, the presence of long-range π conjugation in fluorescent conjugated POPs enables a cascade of energy transfer from the donor polymer to the acceptor molecules, facilitating visible-light-driven photocatalysis [78,82].

Molecular dynamics simulations have been utilized to anticipate and comprehend innovative design ideas and to help determine the structure–property link in POPs. For example, Das et al. reported that porous organic polymer (Py-POP) enhanced with pyridine and coupled with imines can effectively convert CO_2_ to cyclic carbonate (CC) when exposed to visible light [72]. Styrene epoxide (STE) served as a model compound for the production of styrene carbonate (STC) in the presence of *tert*-butyl ammonium bromide (TBAB). The pyridinic N atom in Py-POP allows the photogenerated electrons to move through porous conjugated channels and transfer to CO_2_, activating CO_2_. The effectiveness of the reaction was increased under moderate reaction conditions by photoactivated CO_2_*, which functions more effectively than inactivated CO_2_.

### 5.3. Recent Breakthroughs in Metal-Free POPs for CO_2_ Reduction

In 2020, Yuxi Hou et al. developed two porphyrin-based organic polymers connected by azo bridges through coupling processes. These POPs demonstrated exceptional photocatalytic activity, negating the need for metal co-catalysts or sacrificial reagents in the reduction of CO_2_ to CO. The strong electrostatic contact between CO_2_ and the porphyrin POP, linked by BPY (bipyridine), resulted in the exceptional photo reduction of CO_2_, with 100% selectivity and strong sustainability [66]. In 2022, Subhash Chandra Shit et al. [83] developed a novel metal-free donor–acceptor (D-A) porous polyimide photocatalyst called PeTt–POP using a catalyst-free one-pot polycondensation technique. When exposed to visible light in a gas–solid environment, PeTt-POP demonstrated the ability to convert CO_2_ into CH_4_ without the use of co-catalysts or sacrificial agents, producing 125.63 ppm g^−1^ of CH_4_ in 6 h [83].

Two metal-free amorphous POPs, a carbazole derivative of terpyridine 9-(4-([2,2′:6′,2″-terpyridin]-4′-yl) phenyl)-9H-carbazole (CzTPY) monomers and 1,2,3,5-tetrakis(carbazol-9-yl)-4,6-dicyanobenzene (4CzIPN), were reported recently by Xinyue Hong and colleagues [36]. These were created for highly efficient and selective photocatalytic reduction of CO_2_ in 100% aqueous solution without needing external photosensitizers or catalysts. Even with water as the only solvent, the selectivity remained at 94.7%, suggesting that it could be combined with photocatalytic processes for CO_2_ reduction and H_2_O oxidation. The photocatalysts also showed outstanding stability and recyclability during photocatalysis. Most importantly, they demonstrated a strong capacity for CO_2_ reduction and high CO2 adsorption, providing a viable method for simultaneous capture and use of CO_2_ under moderate conditions with low energy consumption when powered by light [36].

A 2D triazine-based metal-free COF (Tta-TFPA) has a bandgap energy of 1.86 eV and was designed to produce solar fuel efficiently through CO_2_ reduction [84]. The COF (Figure 7) demonstrated excellent light-induced photocatalytic performance, generating formic acid and methanol from CO_2_ under visible light at atmospheric pressure. Particularly, Tta-TFPA COF produced formic acid, as the main product at a rate of 48 mol g^−1^ h^−1^, and methanol, as a minor product at a rate of 8.3 mmol g^−1^ h^−1^, through the CO_2_ reduction reaction [84].

In 2023, Narzary, et al. [85] developed bifunctional metal-free porous polyimide (PPIs) networks for CO_2_ capture and conversion. In this work [85], two perylene-based PPIs were successfully synthesized via polycondensation reactions. The synthesized material exhibited notable porosity and CO_2_ uptake capabilities of up to 4.9 wt%. These PPIs were effectively utilized for CO_2_ capture and as heterogeneous catalysts for CO_2_ utilization. Both PPI-1 and PPI-2 exhibited outstanding recyclability and excellent catalytic performance for synthesizing cyclic carbonates from CO_2_ and epoxides, achieving up to 98% conversion without solvents or co-catalysts. In 2022, Mondal et al. [86] successfully created a metal-free porous polyketone (TPA-DPA PPK) with donor–acceptor (D-A) groups and extensive π-conjugation. This was achieved through a simple Friedel–Crafts acylation reaction between triphenylamine (TPA) and pyridine-2,6-dicarbonyl dichloride (DPA). Notably, TPA-DPA PPK can act as a metal-free catalyst without the need for any additional cocatalyst or sacrificial agent. Under the same conditions, CH_4_ production (152.65 ppm g^−1^) was about five times higher than that of g-C_3_N_4_ [86].

On the other hand, recently, [87] reported that metal-free amine-incorporated triazine-based polymers exhibited remarkable efficiency in converting CO_2_ into cyclic carbonates [87]. The synthesis of the polymer (CTP-X-NH_2_ with X being one or two) utilized 4,6-dichloro-1,3,5-triazine-2-amine (Tz-NH_2_) as the primary precursor and either BP or TPB as co-precursors or linker units. Among various cocatalysts, n-Bu4NBr exhibited a synergistic effect on the cycloaddition of CO_2_ to epoxides. Due to the higher density of amine groups in CTP-1-NH_2_, the CTP-1-NH_2_/n-Bu_4_NBr combination displayed superior catalytic conversion compared to CTP-2-NH_2_/n-Bu_4_NBr. Notably, the CTP-1-NH_2_/n-Bu_4_NBr system achieved a high conversion rate (>93%), with a selectivity of 98% for ECC. Additionally, CTP-1-NH_2_ maintained its original catalytic efficiency through six consecutive cycles, indicating its outstanding stability. Furthermore, CTP-1-NH_2_ effectively facilitated the CO_2_ cycloaddition reaction with various epoxides. Remarkably, the CTP-1-NH_2_/n-Bu_4_NBr system outperformed numerous amine- or nitrogen-containing POPs in cycloaddition reactions conducted at low temperatures and pressures.

A new porous polyimide photocatalyst, PeTt–POP, utilizing a donor–acceptor (D–A) system (perylene dianhydride (Pe) as a donor and 4,4′,4″(1,3,5-triazine-2,4,6-triyl)trianiline (Tt) as an acceptor), monomers, was crafted through a catalyst-free one-pot polycondensation process [83]. PeTt–POP could produce CH_4_ (125.63 ppm g^−1^ in 6 h) from CO_2_ when exposed to visible light in the gas–solid phase, obviating the requirement for co-catalysts or sacrificial agents. Benefiting from robust visible light-absorption capabilities, a well-suited energy band structure, and carbonyl and triazine functional groups serving as effective adsorption sites for reactants and intermediates, PeTt–POP exhibited commendable performance, yielding a 2.6 times enhancement of CH_4_ production compared to gC_3_N_4_ [83].

Yuhan Liu and Yue Wang have developed a new metal-free heterostructure photocatalyst. They used photo-reduced graphene oxide and triazine-based COF (PRGO/TP-COF) to enhance photocatalytic CO_2_ reduction. The PRGO component produces hot electrons that help separate charge carriers with the help of infrared (IR) heat. A 25% PRGO/TP-COF composite achieved an impressive CO yield of 48.81 μmol/g due to the combined effect of PRGO’s photothermal properties and the heterojunction between PRGO and TP-COF [88].

Furthermore, a new triazine-based COF (COFTVBT-N) was efficient for the photoreduction of CO_2_ to HCOOH under simulated sunlight without the need for metal sites or photosensitizers. COFTVBT-N demonstrated excellent photocatalytic performance, achieving a high HCOOH production rate of 440.6 μmol⋅g^−1^⋅h^−1^. The adsorption of H_2_ on the triazine rings of 2,4,6-tris(4-vinylbenzoyl)-s-triazine (TVBT) enhances the stability of CO_2_ adsorption, while CO_2_ reduction is facilitated by the synergistic effect of the alkenyl groups [89].

The other triazine-based two-dimensional COF (TRITER-1) was synthesized via the Schiff-base condensation reaction between terephthalaldehyde and 1,3,5-tris(4-aminophenyl)triazine. Incorporating g-C_3_N_4_ into this imine-based COF resulted in the composite TRITER-1@g-C_3_N_4_, which exhibited a relatively small band gap of 2.0 eV. Under a 15-W white LED irradiation by visible light, the maximum yield (TON = 172) of CO_2_ reduction to CH_3_OH (major product) was achieved utilizing only 10 mg of crystalline TRITER-1@g-C_3_N_4_ composite. The catalyst was recycled several times without losing its catalytic performance [90].

In 2023, Xiao et al. reported a novel, hydrophilic, and fully conjugated COF for photocatalytic CO_2_ reduction to CO using nearly pure water (QL-COF) (Figure 7). Converting imine linkers into 4-carboxyl-quinoline linkages in COFs enabled the development of efficient, crystalline porous polymeric photocatalysts for CO_2_ reduction using H_2_O as the electron donor. In addition, it showed excellent activity and photostability for the photoreduction of CO_2_, with a high selectivity of 99.3% for CO generation (156 mmol g^−1^ h^−1^) under simulated sunlight irradiation [91].

A recent study [92] reported that an ethene-based COF has shown remarkable efficiency as a metal-free photocatalyst for converting CO_2_ to methane under visible light. The COF has excellent crystallinity and a high specific BET surface area of 1150 m^2^ g^−1^. Moreover, it exhibits superior thermal stability up to 532 °C and an ultrahigh CO_2_ uptake of 112 mg/g at 298 K. It achieved 100% selectivity, with a production rate of 14.7 µmol g^−1^ h^−1^ and an apparent quantum yield of about 0.99% at 489.5 nm. These results are quite promising for CO_2_ conversion by a metal-free COF photocatalyst, especially since it does not require a co-catalyst [92].

Sheng-Yan Yin and his team have developed a new metal-free POP for the efficient room-temperature photocatalytic reduction of CO_2_ through the dry-reforming of methane. This material has enhanced the specific surface area, light-absorption capacity, and photoelectron-generation efficiency. It also significantly enhanced charge-carrier separation, increasing syngas production. After 20 h of illumination, the photocatalytic results indicated that the yields of CO and H_2_ were 1123.6 mmol g^−1^ and 30.8 mmol g^−1^, respectively [93].

## 6. Conclusions

This review has provided an overview of various POPs-based catalysts, especially metal-free photocatalysts used for CO_2_ reduction. Utilization of metal-free POPs can resolve the major problem of high production cost to a large extent. However, several challenges still exist that need to be solved to achieve efficient CO_2_ reduction. POPs may act as an adsorbent for CO_2_ and a photo-induced CO_2_ reduction catalyst at the same time. One of the major challenges that researchers face with the POPs is the inability to attain precise control over the pore size. Porosity is directly linked with adsorption capabilities. POPs have relatively large pore sizes and low surface area. Enhancing the surface area can provide more active sites for redox reactions, but the active recombination of charge carriers can adversely affect the long-term stability of the photocatalyst. POPs need to be developed with simultaneous control over porosity and surface area. While tuning POPs remains a great challenge, modifying the POP’s structure to tune its band gap can shift it to the visible region, enhancing the effective utilization of CO_2_. The direct relationship between the structure of a POP, its catalysis, and its performance in photocatalytic reactions has not been well studied. Comprehending this relationship is impeded by the challenge of precisely monitoring and identifying crucial reaction intermediates. Despite these difficulties, POPs still offer significant advantages in the environmental domain and show immense potential for use in environmental remediation, pollution monitoring, and related applications. We anticipate this study will provide valuable insights for creating and synthesizing functional POPs to enhance their photocatalytic activity for CO_2_ reduction.

## Figures and Tables

**Table 1:** Summary of the reported catalysts used for CO_2_ reduction.

Type of Catalyst	Catalyst	Product(s)	Conversion %	Ref.
Thermocatalyst	Ru- CeO_2_	CH_4_	100%	[9]
	Pt-TiO_2_	CO	100%	[10]
	Pd-In_2_O_3_	CH_3_OH	78%	[11]
	Co-doped ZrO_2_	CO	95%	[12]
	Ni-MgO	CO		[13]
	Ni-CNT/TiO_2_	CO	95.5%	[14]
	Fe_3_O_4_ with Pd	C_2_H_5_OH	0.3%	[15]
	CNT, Pd and La_2_O_3_	CO	36%	[16]
	N-doped C with Ni	CO	51%	[17]
	ZnO with Pd	HCOOH		[18]
Electrocatalyst	Fe–N–C	CO	93%	[19]
	Fe/Pc graphene composites	CO	>94%	[20]
	Co–N_2_ on ZIF	CO	94%	[21]
	Cu-FeSA	CH_4_	64%	[22]
Photocatalyst	Mo-COF	CO	6.19 µmol g^−1^ h^−1^	[23]
		CH_4_	1.08 µmol g^−1^ h^−1^	
	Co-MOF 525	CO	200.6 µmol g^−1^ h^−1^	[24]
		CH_4_	36.67 µmol g^−1^ h^−1^	
	Ni-SA-5/ZrO_2_	CO	11.8 µmol g^−1^ h^−1^	[2]
	POPnFe	CO/H_2_		[25]
	(POP)-embedded In_2.77_S_4_	C_2_H_5_OH	67%	[26]
	S-rich thiacalixarene-based POP with AuNP	CO	6.74 μmol g^−1^ over 4 h	[5]
	LaYAgO_4_-Graphene-TiO_2_	CH_3_OH	12.27%	[3]

## Data Availability

All data are available in the paper.

## Abbreviations

BPY	Bipyridine
C_2_H_4_	Ethene
C_2_H_5_OH	Ethanol
C_2_H_6_	Ethane
CB	Conduction Band
CH_2_O	Formal aldehyde
CH_3_OH	Methanol
CH_4_	Methane
CMP	Conjugated Microporous Polymers
CO_2_	Carbon dioxide
COF	Covalent Organic Framework
CTF	Covalent triazine frameworks
D-A system	Donor–Acceptor system
DFT	Density Functional Theory
EY dye	Eosin Y Dye
H_2_O	Water
HCO_2_H	Formic acid
HOF	Hydrogen-Bonded Organic Framework
HOMO	Highest Occupied Molecular Orbital
LUMO	Lowest Unoccupied Molecular Orbital
PCP	Porous Conjugated Polymers
POP	Porous Organic polymers
PPI	Porous Polyimide
PT-CMPS	Pyrene-4,5,9,10-tetraone-linked Conjugated Microporous Polymers
VB	Valence Band
